# Changes in Cytokines of the Bone Microenvironment during Breast Cancer Metastasis

**DOI:** 10.1155/2012/160265

**Published:** 2012-01-23

**Authors:** Donna M. Sosnoski, Venkatesh Krishnan, William J. Kraemer, Courtenay Dunn-Lewis, Andrea M. Mastro

**Affiliations:** ^1^Department of Biochemistry and Molecular Biology, Penn State University, University Park, PA 16802, USA; ^2^Department of Kinesiology, University of Connecticut, Storrs, CT 06269, USA

## Abstract

It is commonly accepted that cancer cells interact with host cells to create a microenvironment favoring malignant colonization. The complex bone microenvironment produces an ever changing array of cytokines and growth factors. In this study, we examined levels of MCP-1, IL-6, KC, MIP-2, VEGF, MIG, and eotaxin in femurs of athymic nude mice inoculated via intracardiac injection with MDA-MB-231^GFP^ human metastatic breast cancer cells, MDA-MB-231BRMS1^GFP^, a metastasis suppressed variant, or PBS. Animals were euthanized (day 3, 11, 19, 27 after injection) to examine femoral cytokine levels at various stages of cancer cell colonization. The epiphysis contained significantly more cytokines than the diaphysis except for MIG which was similar throughout the bone. Variation among femurs was evident within all groups. By day 27, MCP-1, MIG, VEGF and eotaxin levels were significantly greater in femurs of cancer cell-inoculated mice. These pro-osteoclastic and angiogenic cytokines may manipulate the bone microenvironment to enhance cancer cell colonization.

## 1. Introduction

The colonization and growth of cancer metastases in the bone depends on a cooperative interaction of the cancer cells with the host cells in the bone microenvironment. This microenvironment includes the resident osteoblasts, osteoclasts, endothelial cells, bone-lining cells, stromal cells, hematopoietic stem cells, and transient cells such as macrophages, lymphocytes, neutrophils, and other blood cells. While cell-cell contacts are established between cancer cells and bone cells via adhesion molecules, a wider network of communication occurs through secreted cytokines and growth factors. These soluble molecules play a critical role in the normal bone remodeling process as well as in cancer cell colonization of the bone marrow. 

The interplay of the cancer cells with the cells of the bone marrow cavity has been described in terms of a vicious cycle [1]. In brief, cytokines or growth factors secreted by invading cancer cells (e.g., parathyroid hormone-related protein, PTHrP) act to stimulate osteoblasts to produce more receptor activator of nuclear factor kappa-B ligand (RANKL) and less osteoprotegerin (OPG), a decoy receptor for RANKL. The RANKL binds to RANK on osteoclast precursors leading to differentiation and activation of osteoclasts. Activated osteoclasts degrade bone matrix releasing growth factors such as transforming growth factor beta (TGF-*β*) and insulin-like growth factor (IGF). These molecules, in turn, stimulate further cancer cell growth. This series of events provides an explanation of the osteolytic outcome of breast cancer metastasis in bone; that is, an increase in osteoclast activation leads to excess bone breakdown and further stimulation of cancer cells. Drugs targeted to osteoclasts slow down formation of bone lesions. However, by and large, the lesions do not heal. In our previous research, we found that metastatic breast cancer cells also inhibit the differentiation of osteoblasts, thereby diminishing bone formation. The combination of increased bone degradation and decreased bone rebuilding has a net outcome of bone loss.

Through cell culture studies, we discovered that metastatic breast cancer cells induce an osteoblast inflammatory response. When conditioned medium from metastatic human breast cancer cells, MDA-MB-231, was added to human osteoblasts (hFOB1.19) or murine (MC3T3-E1) or primary osteoblasts, the osteoblasts increased their secretion of interleukin-6 (IL-6), interleukin-8 (IL-8), and monocyte chemoattractant protein-1 (MCP-1). Under these conditions, the osteoblasts did not differentiate in culture; that is, they did not produce characteristic osteoblast differentiation proteins such as alkaline phosphatase, osteocalcin, or bone sialoprotein [2, 3]. 

These observations were followed with *in vivo* experiments. By using green fluorescent protein (GFP) expressing cancer cells in a xenograft model, we were able to monitor the progress of cancer colonization in the femurs [4]. We saw that the cancer cells appeared throughout the bone but cleared quickly from the diaphysis. Some cells localized in the ends of the femur where they developed into large colonies. For the most part, the cancer cells were associated with the endosteal surface of the bone marrow compartment. As part of an *ex vivo* study, we examined the cytokines produced by the bone cells in the presence of cancer cells [5]. Mice received intracardiac injections of MDA-MB-231^GFP^ cells and were sacrificed three weeks later. In this study, the marrow was removed from the femurs, the bones separated into diaphysis (shaft) and epiphyses (ends), crushed and incubated in culture medium for 24 hr. Species-specific antibodies were used to distinguish between host (murine) and cancer (human) cytokines. We found that murine IL-6, MCP-1, macrophage inflammatory protein-2 (MIP-2) (human IL-8), vascular endothelial growth factor (VEGF), and keratinocyte chemoattractant (KC) (human growth-regulated oncogene-alpha, GRO-*α*) were greater in ends of the bone compared to shafts and were increased in cancer-bearing mice. This *ex vivo* assay confirmed the *in vitro* findings that host cytokines in the bone microenvironment increase in the presence of cancer cells. 

These initial findings led us to investigate how the cytokine profile of the bone microenvironment changed over time following the appearance of cancer cells in the bone marrow. We designed an experiment to ask how cytokines changed over time in the femurs of mice inoculated with metastatic MDA-MB-231^GFP^ cells. Concurrently, we wished to investigate whether or not the cytokine profile of the bone microenvironment differed when the mice were injected with the highly metastatic MDA-MB-231 line or the metastasis-suppressed variant MDA-MB-231BRMS1 which traffics to the bone but does not grow there [6]. In order to examine the bone microenvironment in its entirety, the bone marrow was left intact. Femurs were separated into shafts and ends, crushed and incubated for 24 hours in serum-free medium. An initial assay panel of 32 mouse cytokines revealed two cytokines, eotaxin and monkine induced by interferon gamma (MIG), in addition to IL-6, MCP-1, MIP-2, KC, and VEGF that merited further investigation. In the final experiment, athymic nude mice were injected in the left cardiac ventricle with either MDA-MB-231^GFP^ cells, MDA-MB-231BRMS1^GFP^ cells, or PBS. Four days were chosen for sacrifice (3, 11, 19, and 27 days after injection) to represent early, middle, and late stage metastasis. Culture supernatants from femoral shafts and ends were analyzed for MCP-1, IL-6, KC, MIP-2, VEGF, MIG, and eotaxin. Changes in cytokine levels were compared over time as well as between injection groups.

## 2. Materials and Methods

### 2.1. Cell Lines

The human metastatic breast cancer cell line MDA-MB-231^GFP^ (231) and the metastasis-suppressed derivative MDA-MB-231BRMS1^GFP^ (BRMS1) were obtained from Danny Welch, University of Alabama, Birmingham and cultured in DMEM (Mediatech, Herndon, VA), 5% fetal bovine serum (PAA Laboratories, Etobicoke, ON, Canada), and 1X nonessential amino acids (Mediatech). Antibiotics were not used to culture cells for a minimum of two weeks prior to injection. For intracardiac injection, cells were detached with trypsin-EDTA solution, centrifuged and washed twice with sterile phosphate-buffered saline (PBS, Hyclone, Logan, Utah). Cells were resuspended at a concentration of 1.5 × 10^6^ cells/mL in sterile PBS and held on ice until injection.

### 2.2. Intracardiac Inoculation

Six-week-old female athymic nude mice were obtained from Charles River Laboratories and were housed and handled in strict accordance with IACUC regulations (Penn State IACUC Protocol 28631). On the day of inoculation, mice were anesthetized with 120 mg/kg body weight of ketamine and 16 mg/kg of xylazine. When animals were completely anesthetized, 200 *μ*L of PBS or cancer cell suspension (3 × 10^5^ cells) were injected directly into the left ventricle of the heart. For the pilot experiment to screen for relevant cytokines, 3 mice were injected with either PBS, 231, or BRMS1-expressing cells and kept for a period of 3 weeks before sacrifice. For the primary experiment, 8 mice were inoculated with either PBS, 231, or BRMS1-expressing cells for each of the four time points. After recovery from the procedure, mice were returned to sterilized cages with air filters and observed daily for signs of illness or distress. On days 3, 11, 19, and 27 after injection, mice were euthanized by CO_2_ inhalation. Both femurs were removed from each mouse, cleaned of exterior tissue, and placed in PBS on ice prior to processing.

### 2.3. Fluorescence Stereomicroscopy and Metastasis Detection

Femurs were examined by fluorescence stereomicroscopy (40x magnification) with a Nikon SMZ 1500 Fluorescence Stereoscope (Nikon Instruments, Inc., Melville, NY) with GFP long bandpass fluorescence filter (excitation = 488 nm; emission = 515 nm, Chroma Technology Corporation, Rockingham, VT). Images were captured using a Nikon Coolpix 8400 digital camera (Nikon Instruments, Inc.).

### 2.4. Femur Cultures

Proximal and distal ends of each femur were separated from the shaft of the bone. The ends were cut so that they contained the epiphyseal plates and the metaphyses. The ends were placed together in a 2 cm^2^ tissue culture well. The shaft was placed in a separate well. Bone samples were crushed with a small glass pestle, and the fragments were cultured in 1 mL of *α* MEM (MediaTech) for 24 hours. Culture supernatants were then collected, centrifuged to remove cells and bone fragments, and frozen at −80°C.

### 2.5. Cytokine Assays

To determine which cytokines might play an important role in the metastasis of cancer to bone, we first assayed bone culture supernatants of femurs of mice that had been injected with either PBS, 231, or BRMS1-expressing cells 3 weeks prior to sacrifice. For this determination, we used a Milliplex 32-plex mouse cytokine array (Millipore Corporation, Billerica, MA) which allowed for the simultaneous quantitation of 32 mouse cytokines. After relevant cytokines were established for the main experiment, eotaxin, KC, MIG, MIP-2, and VEGF were assayed using a Milliplex 5-plex mouse cytokine array. Two other cytokines, IL-6 and MCP-1 were assayed by standard sandwich ELISA techniques as previously described [7].

### 2.6. Statistical Analysis

A statistical model was fit to allow for correlations within a mouse and within batches of mice using random effects in order to compare each cytokine across each group, that is, PBS, MDA-MB-231, and MDA-MB-231BRMS1. Prior to statistical analyses, assumptions for linear statistics were verified and log_10_ transformations were used and rechecked to assure statistical validity of the analyses. For the shaft versus ends comparison, pg/mL values were analyzed with a two-way ANOVA analysis with a Bonferroni post hoc correction. To compare each cytokine among injection groups and over time, a two-way ANOVA test with a Fisher's LSD post hoc analysis was used. Shown in bar graphs is the mean ± standard error (SE). Box plots display mean, 25–75 percentile range and max/min values. Significance was set at *P* ≤ 0.05.

## 3. Results

### 3.1. Selection of Cytokines

In previous experiments, we had access to a limited panel of cytokines available in a multiplex format. In preparation for this current study, we carried out a more extensive screen with a 32-plex cytokine array to identify more cytokines of interest. Femurs were harvested from 9 mice (3 per group) three weeks following intracardiac inoculation with MDA-MB-231^GFP^ cells, MDA-MB-231BRMS1^GFP^ cells, or PBS. Two femurs from each mouse were incubated as described in the methods section and the culture supernatants were tested. The patterns of cytokines were similar in all three groups but generally highest in the culture supernatants from MDA-MB-231 cells. Of the 32 murine cytokines tested (Table 1), nine were below the levels of detection, approximately 3.2 pg/mL. Another four (IL-2, IL-17, M-CSF, and RANTES) were present in very small amounts, ≤10 pg/mL. Thirteen ranged in concentrations from 10 to 100 pg/mL. Six (G-CSF, IL-6, KC, MCP-1, MIG, and VEGF) were present at >100 pg/mL. We had previously reported that IL-6, KC, MCP-1, VEGF, and MIP-2 were secreted by osteoblasts and increased in the presence of breast cancer cells *in vitro* and *in vivo* [5]. We choose to assay for these five cytokines plus MIG which ranged in concentration from 100–1000 pg/mL. MIG is a target gene of RANKL and is involved in osteoclast activation [8]. We also selected eotaxin (10–40 pg/mL) because of its reported roles in angiogenesis in breast cancer metastasis patients [9] and in multiple myeloma [10]. The multiplex cytokine array for the remainder of the study included IL-6, KC, MCP-1, VEGF, MIG, MIP-2, and eotaxin. At the conclusion of the study, it was found that MIP-2 levels were negligible for most of the samples assayed and were not considered in further analyses.

### 3.2. Detection of Femur Metastases

**Figure 1:**
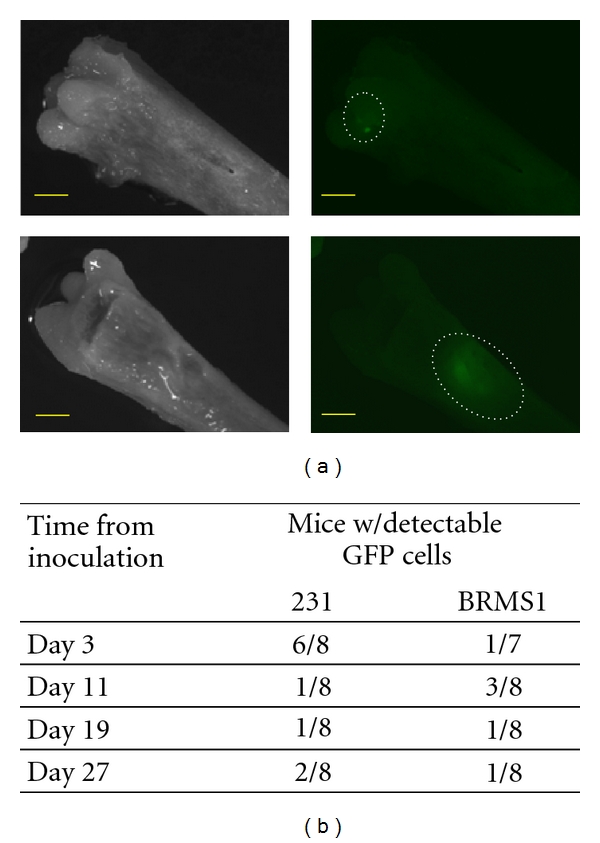
Breast cancer metastases in femoral bone. Female athymic nude mice were inoculated in the left cardiac ventricle with 3 × 10^5^ MDA-MB-231^GFP^ or MDA-MB-231BRMS1^GFP^ cells, eight per group. Animals were euthanized at day 3, 11, 19, or 27 after injection. Femurs were removed, placed in PBS, and imaged with light and fluorescence stereomicroscopy at a 40x magnification to detect GFP-labeled cancer cell metastases. Images are shown for 27 day metastases (a) of MDA-MB-231BRMS1 (top) and MDA-MB-231 (bottom). Note colony size difference between the two variants. Scale bar = 1 mm. Table (b) summarizes the incidence of detectable GFP-expressing cells for each injection group.

Prior to crushing the femurs for incubation, they were examined with a fluorescence stereomicroscope. We detected GFP in some of the femurs of mice inoculated with MDA-MB-231^GFP^ or MDA-MB-BRMS1^GFP^ cells taken at various times (Figure 1). However, the sensitivity of the microscope and the location of the cells combined with the thickness of the bone made it likely that not all metastatic cells were detected by microscopy. There were not enough femurs with GFP detectable colonies to be examined as a group for cytokines separate from the other femurs. For the most part, the GFP-expressing metastases appeared much larger in the femurs of mice inoculated with MDA-MB-231^GFP^ than those with MDA-MB-231BRMS1^GFP^ (Figure 1).

### 3.3. Cytokines in the Diaphysis versus the Epiphysis of the Femurs

**Figure 2:**
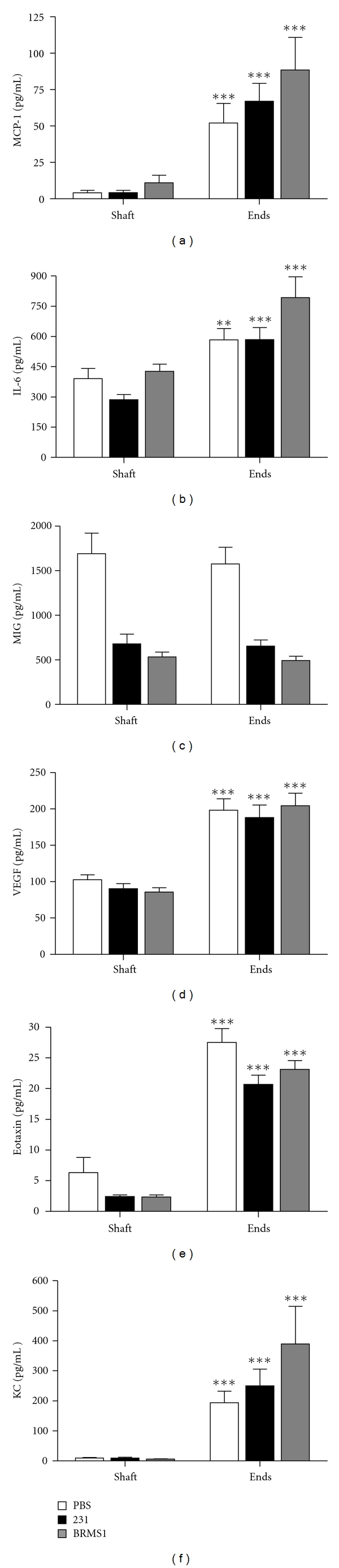
Cytokine levels in bone diaphysis versus bone epiphysis. Athymic nude mice received intracardiac inoculations of either PBS, MDA-MB-231^GFP^, or MDA-MB-231BRMS1^GFP^ cells. Femurs were harvested at days 3, 11, 19, and 27 after inoculation and separated into shafts and ends. Shown here are the results from day 19, but the results were similar for the other days. Bone sections were crushed and cultured in serum-free medium for 24 hours. Resulting supernatants were assayed for MCP-1 (a), IL-6 (b), MIG (c), VEGF (d), eotaxin (e) and KC (f). MIP-2 values were very small or below the level of detection and were not included. With the exception of MIG, the cytokine levels were significantly higher in the ends of the femur than in the shaft. ***P < 0.001; **P = 0.01–0.001. *n* = 8 for each group.

We first compared the levels of MCP-1, IL-6, MIG, KC, VEGF, and eotaxin in the diaphysis (shaft) versus the epiphyses (end) of each bone. The epiphyseal end of the femur contains the metaphysis, the region of bone remodeling rich in cytokines and growth factors, in addition to the epiphyseal growth plate. In contrast, the function of the diaphysis is to provide support and is less metabolically active. As expected, the ends of long bone were a much richer source of cytokines than the shaft (Figure 2). The exception was MIG which was found distributed equally in both areas. Eotaxin was present in less than 10 pg/mL in the shaft supernatant but was 5-6- fold higher in the ends. MCP-1 was also present in low concentrations but ends contained about 10-fold more. Little to no KC was found in the shaft but approximately 200 to 600 pg/mL were detected in the ends depending on the group of mice. VEGF and IL-6 were present in the shafts at about half of the concentration in the ends of the bone which was approximately 200 and 600 pg/mL, respectively. The cytokine found in the greatest concentration was MIG registering 500–2000 pg/mL for both shafts and ends.

### 3.4. Comparison of Cytokines in Three Groups of Mice

**Figure 3:**
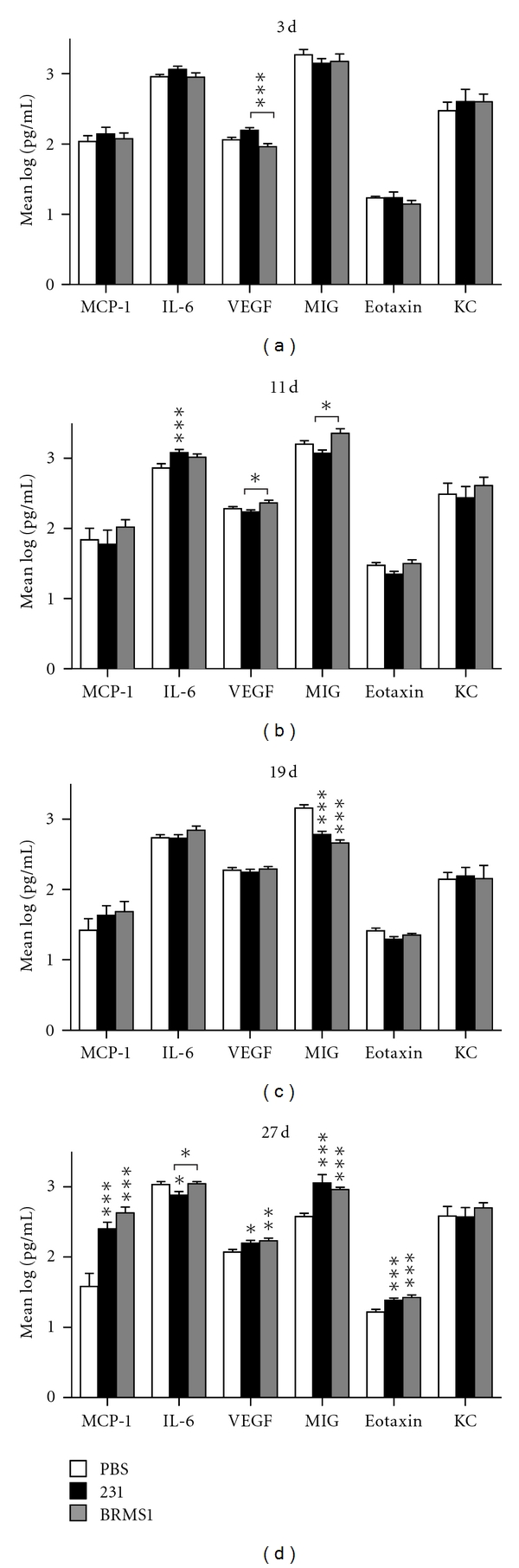
Comparison of bone cytokine levels in mice inoculated with PBS, MDA-MB-231^GFP^ or MDA-MB-231BRMS1^GFP^, cells. Mice were inoculated and femurs processed as described in Section 2. Cytokine values were log_10_ transformed for analysis and graphic comparison of each postinjection group. Statistical significance is shown for comparison to PBS unless otherwise noted with a bracket. The only significant difference shown on day 3 post injection was for VEGF which was higher in 231-injected mice than in BRMS1. On day 11, VEGF and MIG were slightly higher in BRMS1 injected mice than in 231, while IL-6 values were higher in 231 injected mice than in PBS. On day 19, the only cytokine that varied significantly was MIG, with higher values for 231 and BRMS1 mice than in the control animals. In later stage metastasis (day 27), the levels for 4 (MCP-1, VEGF, MIG, and eotaxin) of the 6 cytokines were significantly higher in both the 231-and the BRMS1-injected mice when compared to PBS. IL-6 levels were lower in animals injected with 231 cells. KC levels did not vary between groups at any of the time points. ***P < 0.001; **P = 0.01–0.001; *P = 0.05–0.01. *n* = 8.

The cytokines from femurs of animals inoculated with MDA-MB-231^GFP^ cells, with MDA-MB-231BRMS1^GFP^ cells, or with PBS were compared at four times, day 3, 11, 19, and 27. The values presented (Figure 3) are the cytokine concentrations from the ends of the bone. At the earliest time (day 3), most of the cytokine concentrations were similar in all groups except for VEGF. The femurs of mice inoculated with MDA-MB-231^GFP^ showed significantly greater amounts of VEGF than the mice inoculated with MDA-MB-231BRMS1^GFP^ cells. However, neither group was different than PBS. At day 11, IL-6 was greater in the femurs of mice with MDA-MB-231^GFP^ than in the femurs of the PBS group. Interestingly, at this time, the femurs of the mice inoculated with BRMS1-expressing cells had a greater concentration of VEGF and MIG than the mice with 231 cells. The measurements on day 19 showed few differences among the groups except for MIG. MIG was significantly less in the animals injected with cancer cells than those injected with PBS. By day 27, the differences among groups were most pronounced. MCP-1, MIG, eotaxin, and VEGF were all significantly greater in the cancer-inoculated mice than in those inoculated with PBS. Mice bearing MDA-MB-231^GFP^ showed less IL-6 than those with PBS or MDA-MB-231BRMS1^GFP^ cells. No differences were apparent among the groups for KC at any of the times tested.

### 3.5. Changes in Cytokines over Time

**Figure 4:**
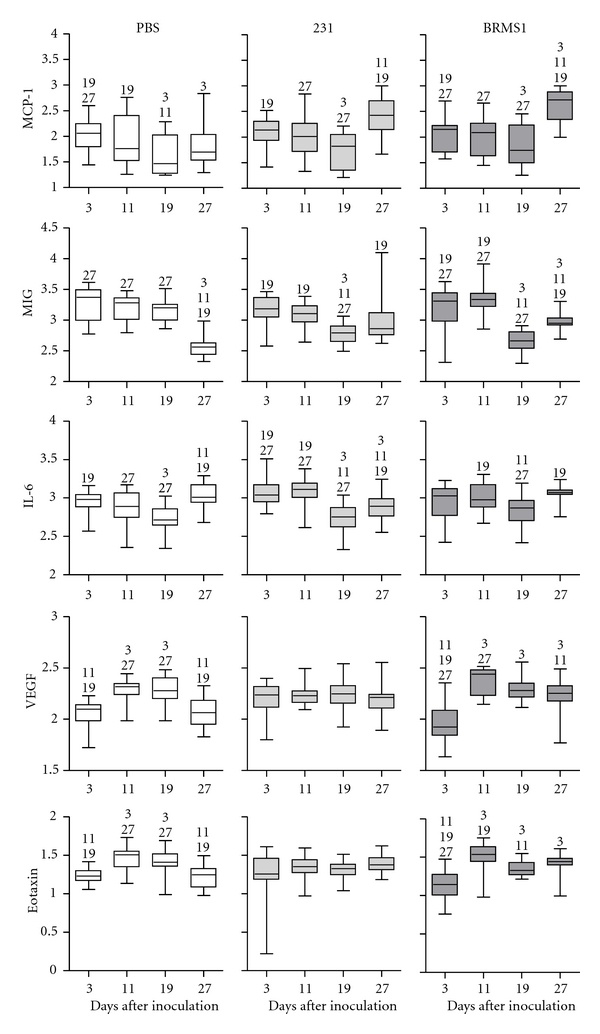
Changes in bone cytokine levels over time. Cytokine values were obtained as described in Section 2. Results were log_10_ transformed for analysis and graphic comparison of each injection group over the 4 points of the study. Each box plot graph represents the change in a particular cytokine level for one injection group over time. The box represents the 75–25% range of values; the horizontal line within the box denotes the mean. Bars above and below the box mark the maximum and minimum values. Numbers above each box denote a significant difference (*P* < 0.05) between time points. *n* = 8.

One of the original objectives of this study was to examine the pattern of cytokine changes over time. We found that there was considerable variation from femur to femur even within the same animal. In the animals treated with PBS, there were increases and decreases over time in 5 cytokines tested (Figure 4). Since these animals did not harbor tumor cells, these differences likely reflect normal physiological variation over time. For the mice inoculated with MDA-MB-231^GFP^ cells, neither VEGF nor eotaxin showed significant increases or decreases over the experimental time frame (Figure 4). In contrast, MCP-1, MIG, and IL-6 exhibited a significant decrease on day 19 when compared to day 3. While IL-6 and MIG levels rose moderately on day 27, the level of MCP-1 was substantially elevated. In animals injected with the metastasis suppressed variant, MDA-MB-231BRMS1^GFP^, the expression pattern for MCP-1, MIG, and IL-6 was similar to results obtained for the metastatic cells. Most notably, MCP-1 levels were significantly elevated by day 27. Interestingly, the BRMS1-expressing cells elicited a variable expression pattern for VEGF and eotaxin that closely resembled the control PBS injection, suggesting that these two cytokines may be implicated in tumor cell colonization. KC was excluded from this analysis due to the lack of change among groups.

## 4. Discussion

Previously, we have reported changes in the inflammatory cytokines IL-6, MCP-1, VEGF, KC, and MIP-2 in the culture supernatants from femurs of athymic mice three weeks after intracardiac injection of MDA-MB-231 cancer cells [5]. We sought to verify and expand these findings to answer several key questions. What other cytokines and growth factors may be involved in the metastatic process? How does the inclusion of the marrow affect the assay of cytokine expression in the presence of metastatic cancer? Does the cytokine profile of the bone microenvironment change over time after the introduction of cancer cells? Does the presence of metastasis-suppressed breast cancer cells elicit a bone cytokine profile that differs from the profile generated by metastatic cancer cells?. 

Cytokine analysis of bone culture supernatants with an expanded 32-plex array revealed the presence of several cytokines in addition to the five (IL-6, MCP-1, VEGF, KC, and MIP-2) previously reported. IL-2, IL-17, M-CSF, and RANTES were detected but only in small amounts; due to cost constraint, we elected not to include them in the panel. MIG and eotaxin were found to be expressed in the mouse femurs and appeared to vary with the presence of MDA-MB-231. MIG is a target for RANKL [8] and as such is involved in osteoclast activation. Eotaxin is believed to play a key role in angiogenesis [11]. Because osteolysis and tumor angiogenesis are intimately tied to cancer metastasis in bone, MIG and eotaxin were included in the cytokine analysis panel.

The epiphyses is a favored site of breast cancer metastasis to bone [12]. Unlike the bone shaft, the ends of the long bones are areas of high bone turnover and are comprised of a specialized arrangement of osteoblasts, osteoclasts, stromal cells, hematopoietic cells, and endothelial cells. In order to examine the cytokine profile of the total bone microenvironment, we left the bone marrow intact when culturing the bones. One obvious outcome of this study was that the cytokine concentrations in the ends of the bones were significantly higher than in the shaft. The exception was MIG. MIG is a product of T cells and endothelial cells. It has also been reported to be produced by osteoblasts [13]. Because the femurs are from athymic mice, the sources of MIG in these experiments are likely osteoblasts and bone endothelial cells. Eotaxin is also a product of T cells and endothelial cells, but not osteoblasts [14, 15]; thus, its likely origin is the bone vascular endothelium. Athymic mice lack T cells but retain much of the innate immune system. We cannot rule out that cytokines may also be due to transient monocytes, or to hematopoietic stem cells. Using 8 mice per injection group per time point, we noticed a great deal of variation in cytokine levels from mouse to mouse within the same time and injection group. In many cases, two femurs from the same mouse yielded very different results. Some of this variation may be due to imprecise sectioning of the ends and shaft of the bones. In addition, transient cells in the marrow such as monocytes or granulocytes and the general health of the animal independent of the presence of cancer metastasis could also account for this wide variation. In the case of animals injected with cancer cells, only the femurs were examined for the presence of metastases. If the cancer cells had colonized another locus in the body, it is possible that the tumor may have had a more widespread effect on the cytokine levels in general.

In order to examine the changes in MCP-1 IL-6, KC, MIP-2, VEGF, MIG, and eotaxin levels over time, we chose to sacrifice animals at 3, 11, 19, and 27 days after injection of MDA-MB-231^GFP^, MDA-MB-231BRMS1^GFP^, or PBS. These times represent early, middle, and late stage metastasis. Unfortunately, this experimental design did not allow us to sample the same animal over time. For this study, two cytokines, MIP-2 and KC, were omitted from final analysis. MIP-2 was not detected at all in a large number of the samples and KC showed no significant changes over time in any of the injection groups. At day 3, 11, and 19, there were some statistically significant changes in MCP-1, MIG, VEGF, eotaxin, and IL-6. Since many of these changes also occurred in mice injected with PBS, the variation can likely be attributed to cyclic expression of cytokines in the bone, possibly due to age. Originally, we postulated that if a particular cytokine was elevated early in the metastatic process, it could be acting as a chemoattractant for cancer cells or a catalyst for cancer cell colonization. However, there is insufficient evidence from this experiment to pinpoint such a cytokine from the seven cytokines assayed. The most striking results were observed at day 27 when levels of MCP-1, MIG, VEGF, and eotaxin were significantly higher in mice injected with either breast cancer cell variant than in mice injected with PBS. IL-6, MCP-1, VEGF, and MIG have all been implicated in osteoclastogenesis [8, 16]. An increase in these molecules in the microenvironment in response to cancer cells correlates with increased osteoclast differentiation and activation and thus bone resorption. Osteoblasts have been reported to display an “inflammatory cytokine stress response” to titanium in joint replacements [17] and to bacteria in osteomyelitis [18]. The same cytokine response occurs when breast cancer and likely other epithelial cells invade the marrow cavity. Because several of these cytokines are also expressed by osteoblasts during their normal differentiation and during the bone remodeling process, it is easy to see how the introduction of cancer cells to the bone microenvironment can disrupt both of these important functions. Additionally, VEGF and eotaxin are known promoters of angiogenesis [11, 19] and may be responsible for the vascularization of a newly formed metastatic tumor.

In comparing the cytokine profiles of animals injected with metastatic MDA-MB-231^GFP^ to metastasis-suppressed MDA-MB-231BRMS1^GFP^ cells, we observed that at day 27 both cell types elicited significant elevations in MCP-1, MIG, VEGF, and eotaxin levels. These data indicate that these four cytokines are not likely to be responsible for the inability of the BRMS1-expressing cells to colonize the bone. However, we were intrigued by the difference in cytokine expression patterns over time for VEGF and eotaxin. While VEGF and eotaxin levels remained unchanged in animals injected with 231 cells, the expression levels for PBS- and BRMS1-injected animals showed a similar pattern of significant variation over time (i.e., reduced expression levels at day 3). One possible interpretation of these data is that higher sustained levels of VEGF and eotaxin are enabling the metastatic cancer cells to colonize and thrive in the bone environment. 

It is interesting to note that MDA-MB-231 and MDA-MB-231BRMS1 themselves secrete IL-6, VEGF, IL-8, and GRO-*α* (the human homologues of MIP-2 and KC, resp.) [5]. MCP-1 is made in small amounts and MIG is reported to be absent from the 231 cancer cells [20]. In this study, human cytokines generated by the cancer cells present in the bone were not measured. In addition, the cancer cells have been reported to express receptors to IL-6 [21], MIP-2 [22], KC [22], VEGF [23], MCP-1 [22], and MIG [24]. The mRNA for the receptor for eotaxin was not detected in MDA-MB-231 cells [22]. In a recent publication, MIG was reported to be produced by bone marrow mesenchymal stem cells and enhanced the invasion and motility of MDA-MB-231 cells [24]. In the cross-species xenograft model for breast cancer utilized in this experiment, mouse cytokines can activate human receptors with the exception of IL-6 [25]. Thus the cytokine changes that occur in the microenvironment as a consequence of the cancer cells may also be responsible for the progression of the metastatic tumor. 

In summary, cytokines in the bone microenvironment are critical components for bone remodeling and hematopoietic processes. The presence of cancer cells changes the normal levels of these cytokines which in turn disrupts the homeostatic balance in the bone. Abnormal cytokine levels may also serve to fuel the propagation and further metastasis of breast cancer cells. Whether these changes are limited to the immediate location of the cancer cells or are the result of a systemic effect has yet to be determined.

## Figures and Tables

**Table 1:** Summary of 32-plex mouse cytokine array.

None detected		1–10 pg/mL	10–100 pg/mL		>100 pg/mL
IFN-*γ*	IL-15	IL-2	Eotaxin	IP-10	G-CSF
IL-3	TNF-*α*	IL-17	GM-CSF	LIF	IL-6
IL-4	IL-7	M-CSF	IL-1*α*	LIX	KC
IL-5	IL-10	RANTES	IL-1*β*	MIP-1*α*	MCP-1
IL-12p40			IL-9	MIP-1*β*	MIG
			IL-12p70	MIP-2	VEGF
			IL-13		
